# Interaction Between Chromium Picolinate Supplementation and Strength Training Modifies Cardiomyocyte Relaxation in Obese Rats

**DOI:** 10.3390/biomedicines14061246

**Published:** 2026-05-30

**Authors:** Kiany Miranda, Wagner Muller Estevam, Daniel Sesana da Silva, Késsia Cristina Carvalho Santos, Luisa Martins Simmer, Amanda Rangel Madureira, Suellem Torezani-Sales, Danilo Sales Bocalini, Ana Paula Lima-Leopoldo, André Soares Leopoldo

**Affiliations:** 1 Postgraduate Program in Physiological Sciences, Health Sciences Center, Federal University of Espírito Santo, Vitória 29047-105, ES, Brazilandre.leopoldo@ufes.br (A.S.L.); 2 Postgraduate Program in Physical Education, Physical Education and Sports Center, Federal University of Espírito Santo, Vitória 29075-910, ES, Brazil; 3 Postgraduate Program in Nutrition and Health, Health Sciences Center, Federal University of Espírito Santo, Vitória 29075-910, ES, Brazil

**Keywords:** chromium picolinate, cardiomyocyte relaxation, high-fat diet

## Abstract

**Background/Objectives:** Chromium picolinate [Cr(pic)_3_] supplementation and strength training (ST) have been proposed as strategies to improve metabolic health in obesity; however, their combined effects on cardiac cellular function remain unclear. This study evaluated the impact of Cr(pic)_3_ supplementation associated with ST on body composition, metabolic parameters, cardiac morphology, and cardiomyocyte contractile function in diet-induced obese rats. **Methods:** Male Wistar rats were fed a high-fat diet and allocated into four groups for 8 weeks: obese sedentary (Ob), obese + ST (ObST), obese + Cr(pic)_3_ (ObCr(pic)_3_), and obese + ST + Cr(pic)_3_ (ObSTCr(pic)_3_). Chromium picolinate (80 μg/kg/day) was administered by gavage, and ST was performed using a ladder-climbing protocol three times per week. Nutritional, metabolic, cardiac morphological, and isolated cardiomyocyte contractile parameters were assessed. A significance level of 5% was set for all tests. **Results:** Neither ST nor Cr(pic)_3_, alone or combined, modified adiposity index, glucose tolerance, insulin resistance, lipid profile (except HDL), or cardiac morphology. ST improved maximal load capacity in trained groups, confirming protocol efficacy. HDL levels were higher in the combined intervention group compared with obese sedentary rats. Cardiomyocyte fractional shortening and maximal contraction and relaxation velocities were unchanged among groups. However, the association of ST and Cr(pic)_3_ resulted in prolonged time to 50% relaxation, indicating delayed relaxation kinetics without alterations in contractile performance. **Conclusions:** These findings suggest that Cr(pic)_3_ supplementation does not enhance metabolic adaptations to ST and may adversely affect cardiomyocyte relaxation dynamics in obesity.

## 1. Introduction

Obesity has been linked to structural and functional changes in the cardiovascular system, such as impaired ventricular relaxation, increased myocardial stiffness and altered excitation–contraction coupling [1,2,3,4]. At a cellular level, obesity has been linked to disturbances in calcium handling, oxidative stress and changes in myofilament sensitivity. These factors may contribute to subtle abnormalities in the contractile performance of cardiomyocytes [5,6,7].

Obesity is one of the major global public health challenges, strongly increasing the risk of cardiovascular disease, particularly heart failure with preserved ejection fraction (HFpEF). Diastolic dysfunction is one of the main underlying pathophysiological mechanisms in this context and can occur even in the absence of apparent comorbidities [8]. Recent evidence suggests that abnormal fat distribution, particularly the accumulation of visceral and epicardial adipose tissue, is central to the development of these changes. This promotes chronic inflammation, lipotoxicity and increased myocardial stiffness [9]. These changes impair ventricular relaxation and elevate filling pressures, contributing to the early development of diastolic dysfunction. However, despite advances in our understanding of this relationship, the mechanisms involved, especially in the early stages of obesity or in the absence of other associated conditions, remain incompletely elucidated. Therefore, further research is needed to investigate the impact of obesity on cardiac diastolic function.

Strength training (ST) is widely recommended as a non-pharmacological strategy for improving metabolic health in people with obesity. As well as its well-established effects on skeletal muscle strength and body composition, ST may influence cardiac structure and function through hemodynamic and metabolic adaptations [10,11]. Experimental studies suggest that exercise training can modulate myocardial calcium cycling, antioxidant capacity and contractile performance [11,12]. However, the effects of ST on cardiomyocyte contractility in established obesity remain poorly understood, and existing data suggest only modest cellular adaptations.

Meanwhile, chromium picolinate (Cr(pic)_3_) is widely promoted as a dietary supplement designed to enhance glucose metabolism, insulin sensitivity and body composition [13,14,15]. While some experimental and clinical studies report metabolic benefits, the evidence is inconsistent. Notably, the cardiac effects of Cr(pic)_3_ supplementation are not well understood. Limited data suggest that Cr(pic)_3_ may influence myocardial performance and calcium homeostasis, but findings vary depending on the dose, duration and experimental model used [13,16,17]. Although Cr(pic)_3_ is widely consumed as a metabolic supplement, little is known about how it might interact with the cardiac adaptations induced by ST, particularly with regard to cardiomyocyte function.

To date, no study has examined the effect of Cr(pic)_3_ supplementation on the contractile properties of cardiomyocytes in the context of ST and diet-induced obesity. This interaction is particularly relevant given the widespread use of nutritional supplements by physically active individuals seeking to improve their metabolism. The present study investigated the effects of combining Cr(pic)_3_ supplementation with ST on metabolic parameters, cardiac morphology and cardiomyocyte contractile function in diet-induced obese rats. It was hypothesized that ST would promote adaptive changes in cardiomyocyte contractile dynamics and that Cr(pic)_3_ supplementation could modify these cellular responses.

## 2. Materials and Methods

### 2.1. Animal Care

In this study, 128 Wistar rats, 30 days old, were housed in polypropylene cages with controlled environment, humidity and temperature, and 12 h lighting cycles. All experimental procedures followed the guidelines established in the “Guide for the Care and Use of Laboratory Animals”. The experimental protocol was approved by the Committee of Ethics in the Use of Animals (CEUA) of the Federal University of Espírito Santo, under number 25/2017.

### 2.2. Experimental Protocol

Following a seven-day acclimatization period, the animals were blindly distributed, by stratified randomization based on body weight, into two groups: a standard diet (SD; *n* = 59) or a high-fat diet (HFD; *n* = 59). The SD group received a diet containing 12.3% fat, 57.9% carbohydrates and 29.8% protein (RC Focus 1765, Agroceres^®^, São Paulo, SP, Brazil), and the HFD group received a diet containing 37.6% fat, 44.6% carbohydrates and 17.8% protein (Nutriave Alimentos^®^, Viana, ES, Brazil). Food consumption was standardized at 40 g per day, with leftovers measured after 24 h, and water was provided ad libitum.

**Figure 1:**
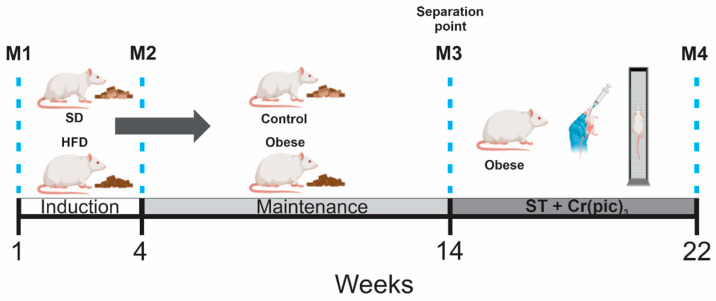
Schematic representation of experimental protocol (22 weeks). M1 = induction of obesity; M2 = maintenance of obesity; M3 = start of treatment; M4 = end of experimental protocol. SD: standard diet (*n* = 59). HFD: high-fat diet (*n* = 59).

The 22-week experimental protocol was divided into four phases: obesity induction (M1; 4 weeks), obesity maintenance (M2; 10 weeks), ST combined with Cr(pic)3 supplementation (M3; 8 weeks), and termination of the protocol (M4) (Figure 1). Initially, obesity was confirmed by a significant increase in body weight in the HFD group compared to the standard group, characterizing the onset of obesity, and they remained that way until the end of M2.

### 2.3. Criteria for the Composition and Redistribution of Groups

At the end of the obesity maintenance phase (M3), with the aim of using homogeneous groups, the groups were refined using the 95% confidence interval (IC95%) based on the final body mass measured during this period. In this way, the separation point between the groups, defined as the midpoint between the upper limit of the SD group and the lower limit of the HFD group, could be defined and then applied as a cutoff point. Thus, animals that were in the SD group but had body mass above the cutoff point, or were in the HFD group and were below the PS, were excluded. At the end, 43 HFD rats and 50 SD rats remained in the study. Because the aim was to evaluate only the effect of CrPic supplementation and/or ST in obese rats. 

To begin treatment, the animals were blindly redistributed again, by stratified randomization based on body weight into four groups according into different groups according to treatment: obese (Ob, *n* = 10); obese submitted to ST (ObST, *n* = 11); obese supplemented with Cr(pic)_3_ (ObCr(pic)_3_ , *n* = 11); obese submitted to ST and supplemented with Cr(pic)_3_ (ObSTCr(pic)_3_, *n* = 11).

### 2.4. Supplementation of Chromium Picolinate (Cr(pic)_3_)

The ObCrPic and ObSTCrPic groups received daily administrations of a chromium picolinate (Chromax^®^, Nutrition 21, Inc., Saddle Brook, NY, USA) solution via orogastric gavage at a dose of 80 μg/kg/day for a period of 8 weeks. This protocol was designed to provide an average daily intake of 8 μg of elemental chromium per rat, which corresponds to approximately 560 μg per day for an adult human weighing 70 kg as described by [18]. To ensure consistent dosing throughout the experimental period, the dosage of CrPic was adjusted weekly based on changes in body weight.

### 2.5. Strength Training Protocol

The resistance training protocol was preceded by a familiarization phase (Week 0), conducted during the final week of the maintenance period (14th week) on three non-consecutive days. During this phase, the animals performed three climbing sets without additional load to adapt to the apparatus (1.1 m × 0.18 m ladder, 80° incline, 2 cm grid spacing, and a resting chamber positioned at the top), which was custom-built by our laboratory.

For load quantification, weights containing iron ore powder were placed in plastic bags and attached to the proximal region of the animals’ tails using adhesive tape. An Initial Maximum Load Test (IMLT) was performed immediately after the familiarization week, prior to the first training week. Maximum Load (ML) was determined starting at 50% of body weight, with increments of 30 g added after each successfully completed climb until failure, with 120 s rest intervals between attempts. To adjust training intensity throughout the experimental period, ML tests were repeated every two weeks (after Weeks 2, 4, and 6), and a Final Maximum Load Test (FMLT) was performed upon completion of the protocol (after Week 8), totaling five assessments [19] (Table 1).

Resistance training was performed three times per week for eight weeks. Each training session consisted of four progressive sets at 50%, 75%, 90%, and 100% of ML. Additionally, the animals performed a fifth set at 100% ML plus an extra 30 g to monitor session-by-session strength progression, with a 60 s rest interval between sets [19].

Training performance was assessed using ML, expressed as both absolute load (g) and relative load, calculated by dividing the absolute load by body weight. Strength progression was determined by the percentage change (Δ) between the final and Initial Maximum Load Tests according to the following equation: Δ(%) = (FMLT − IMLT) × 100/IMLT, where FMLT and IMLT represent the final and Initial Maximum Load Test values, respectively [20].

### 2.6. Nutritional Assessment

The nutritional profile was evaluated by analyzing body weight, body fat and adiposity index. The weight of the animals was measured weekly with the use of an electronic precision scale (Edutec, EEQ9003C). To evaluate the animal’s ability to convert energy from food into body weight, the feeding efficiency (total gain of weight of animals (g)/total energy consumed (Kcal) × 100) was calculated. The amount of body fat was obtained by the sum of visceral fat deposits, known as epididymal (fat adjacent to the epididymis), retroperitoneal (adipose tissue located in the retroperitoneal space and above the kidneys) and mesenteric fat (adipose tissue present in the mesentery). The adiposity index, total body fat (g)/final body weight (g) × 100 was calculated [21].

### 2.7. Biochemical Parameters

The animal’s glycemic profiles were assessed following a 6 h fast in the last week of the protocol. Glycemic levels were measured at baseline and after an intraperitoneal injection of 50% glucose solution (30, 60, 90, and 120 min post injection) [22]. Blood glucose levels were measured using a portable glucometer (Accu-Chek Performa Nano Kit, Roche Diagnostic Ltd., São Paulo, SP, Brazil).

Blood samples were collected in Falcon tubes and centrifuged at 10,000 rpm for 10 min (Heraeus Megafuge 16R Centrifuge, Thermo Scientific, Waltham, MA, USA). Serum concentrations of triglycerides, total cholesterol, low-density lipoproteins (LDL) and high-density lipoproteins (HDL) were determined by specific kits (Bioclin^®^, Belo Horizonte, MG, Brazil) and analyzed with the BS-200, an automated biochemical apparatus. The concentration of insulin (Linco Research Inc, St. Louis, MO, USA) was determined by immunoenzyme assay (ELISA) using a specific kit. The reading was performed using a microplate reader (Asys Expert Plus Microplate Reader, Cambourne, Cambridge, UK). The Homeostatic Model Assessment-Insulin Resistance (HOMA-IR) index was used as the insulin resistance index and calculated according to the formula: HOMA-IR = [fasting blood sugar (mmol/L) × fasting insulinemia (μU/mL)]/22.5 [21]. All analyses were performed blindly.

### 2.8. Cardiac Morphology—Macroscopic Analysis

Cardiac hypertrophy was determined by evaluating the following parameters: total heart weight and its relationship to tibia length. The tibia was removed and cleaned of surrounding soft tissues for subsequent length measurement using a caliper (ZAAS Precision-Amatools Comercial e Importadora Ltd., Piracicaba, SP, Brazil).

### 2.9. Ventricular Cardiomyocyte Isolation and Cellular Contractility

Left ventricular cardiomyocytes were isolated using the retrograde Langendorff perfusion technique. Briefly, the hearts were initially perfused for 2–3 min with a digestion buffer (DB) containing EGTA and HEPES (pH 7.39) to clear the coronary vasculature. Subsequently, enzymatic perfusion was performed using a solution containing collagenase (24 mg) and Ca^2+^ (1 mM) for 10–15 min. All solutions were maintained at 37.5 °C. Following mechanical dissociation, the isolated cells were gradually re-adapted to calcium and maintained in Tyrode’s solution at 37.5 °C, with the following ionic composition (mM): 140 NaCl, 10 HEPES, 0.33 NaH_2_PO_4_, 1 MgCl_2_, 5 KCl, 1.8 CaCl_2_, and 10 glucose.

For the assessment of contractility and relaxation, cardiomyocytes were placed in a glass-bottom experimental chamber and visualized using an inverted microscope (Nikon Eclipse TS100, Melville, NY, USA) equipped with a 40× objective lens. Electrical field stimulation was delivered using a Myopacer stimulator (IonOptix, Milton, MA, USA), with cells stimulated at 1 Hz, 5 ms pulse duration, and 20 V. Approximately 10–15 cells per animal were selected for recording cellular contractility.

Sarcomere length changes were recorded using a 240 Hz camera system (MyoCam, IonOptix) coupled to an edge-detection and data acquisition system (SarcLen, IonOptix, Milton, MA, USA). To ensure methodological reproducibility and data reliability, analyses were restricted to viable cardiomyocytes exhibiting clear cell borders and sarcomeric striations, resting quiescence, and absence of spontaneous contractions.

Functional data were analyzed using IonWizard software (IonOptix). Contractile and relaxation responses were obtained from the averaged overlay of at least six stable contractions. All analyses were performed blindly. The following functional parameters were determined: percentage of shortening (%), maximal shortening velocity (µm/s), maximal relaxation velocity (µm/s), and the time required to reach 50% of peak shortening and 50% relaxation (ms) [23].

During the process of isolating cardiomyocytes from some animals, it was not possible to obtain viable cells for recording contractility; therefore, the sample size was reduced in the following groups: Ob: 5 hearts/74 cells; ObST: 6 hearts/101 cells; ObCr(pic)3: 4 hearts/69 cells; and ObSTCr(pic)3: 7 hearts/98 cells.

### 2.10. Statistical Analysis

The analysis of data distribution was performed using the Shapiro–Wilk normality test. The results of the data were presented by mean and standard error of the mean (SEM). Comparisons between SD and HFD, or C and Ob were conducted using Student’s *t*-test for independent samples. For comparison between Ob, ObST, ObCr(pic)_3_ and ObSTCr(pic)_3_ groups, two-way ANOVA followed by Bonferroni’s post hoc test was used. A significance level of 5% was set for all tests. Statistical analyses and graphical representations were performed using GraphPad Prism 9.0 software (GraphPad, San Diego, CA, USA).

## 3. Results

**Figure 2:**
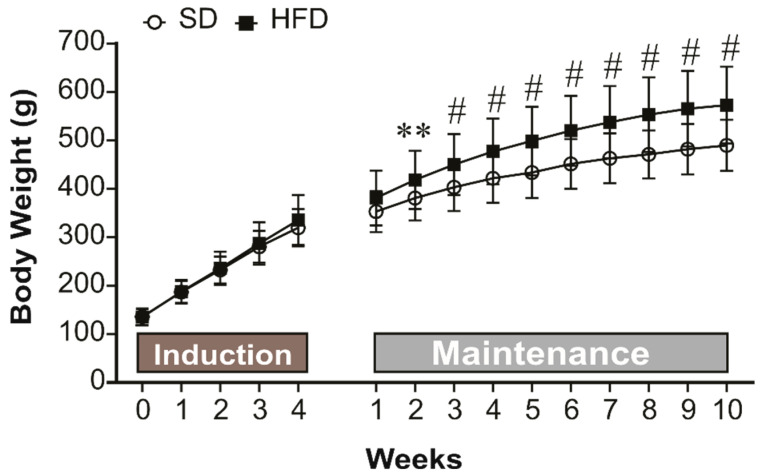
Evolution of body weight during the protocol of induction and exposure to obesity after 14 weeks. Standard diet (SD, *n* = 59), high-fat diet (HFD, *n* = 59). Data are reported as mean ± SEM. Statistical comparisons were performed using two-way ANOVA followed by Bonferroni’s post hoc test. Data were considered significant when ** *p* = 0,01; # *p* < 0.0001.

To validate the experimental model, we analyzed the progression of body weight, body composition, and systemic markers. Rats in the HFD group showed a progressive increase in body weight compared to the SD group from the second week of maintenance, persisting throughout the 14-week period (Figure 2). Despite consuming less feed, rats in the HFD group showed higher caloric intake due to the higher energy density of the diet, accompanied by greater feed efficiency. Consequently, animals in the HFD group had an increase in final body weight and total weight gain (Table 2).

After the 14-week, the animals were redistributed into other groups to continue the experimental protocol. For this analysis, we only evaluated animals from the control and sedentary obese groups in order to characterize the obesity model. Even after the experimental redistribution, as observed in Table 2, rats in the Ob group showed an increase in final body weight (FBW), body weight gain, epididymal, retroperitoneal and visceral fat deposits, body fat, and adiposity index, confirming the induction of obesity by the HFD diet. Additionally, animals in the Ob group demonstrated a systemic increase in fasting blood glucose; however, systemic levels of cholesterol, LDL, and HDL did not differ between the experimental groups (Table 3).

**Figure 3:**
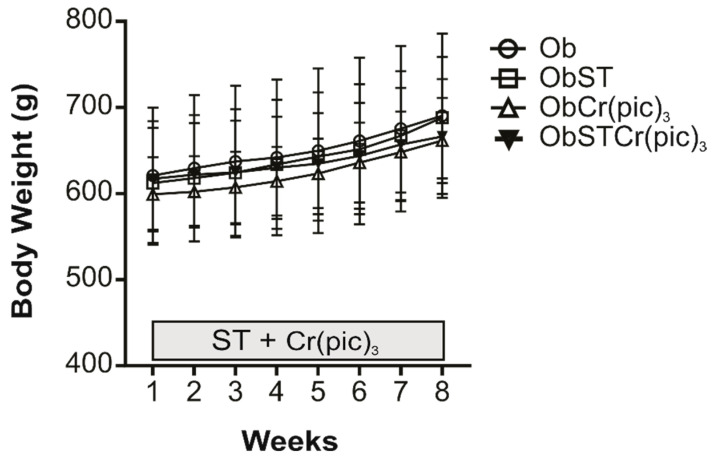
Evolution of body weight during the 8-week treatment period. Animals per experimental group: obese (Ob, *n* = 10), obese strength training (ObST, *n* = 11), obese supplementation with chromium picolinate (ObCr(pic)_3_, *n* = 11), and obese combination of both (ObSTCr(pic)_3_, *n* = 11). Data are reported as mean ± SEM. Statistical comparisons were performed using two-way ANOVA followed by Bonferroni’s post hoc test.

Following the induction of obesity, the animals were redistributed and subjected to an eight-week intervention period to determine whether ST, Cr(pic)_3_ supplementation, or a combination of the two could modify the established obese phenotype. During this period, no differences were observed in body weight (Figure 3), adiposity index, total fat mass or fat deposits between the groups (Table 4). Furthermore, food consumption and caloric intake, as well as feed efficiency, also remained similar between the groups. These findings indicate that established obesity is resistant to short-term interventions, revealing a dissociation between functional improvements and metabolic adaptations.

In line with the lack of changes in body composition, the interventions had little effect on metabolic parameters. Fasting glucose, insulin and HOMA-IR did not differ between groups at the end of the protocol. Total cholesterol, LDL and triglycerides were also unchanged. An exception was observed for HDL concentrations, which were higher in the ObSTCr(pic)_3_ group than in obese sedentary animals. Overall, these data suggest that the interventions produced minimal effects on systemic metabolic variables (Table 5).

**Figure 4:**
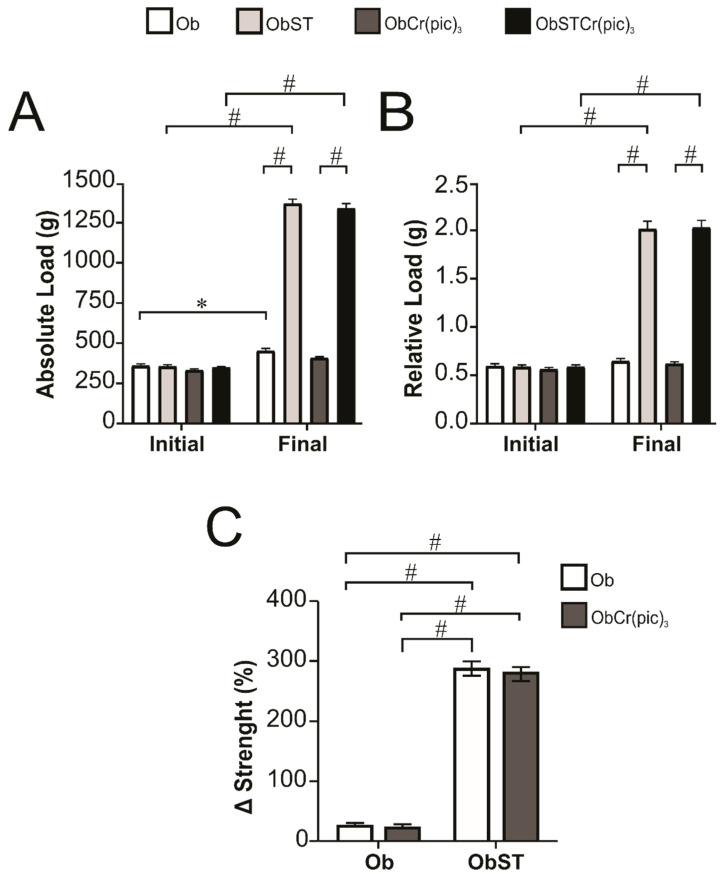
Strength performance of the groups in the absolute load-carrying test and the relative load-carrying test. (**A**) Absolute load. (**B**) Relative load. (**C**) Delta ∆ force training. Data are reported as mean ± SEM. Animals per experimental group: obese (Ob, *n* = 9), obese strength training (ObST, *n* = 11), obese supplementation with chromium picolinate (ObCr(pic)_3_, *n* = 10), and obese combination of both (ObSTCr(pic)_3_, *n* = 11). Statistical comparisons were performed using two-way ANOVA followed by Bonferroni’s test for multiple comparisons. Data were considered significant when * *p* < 0.05. # *p* < 0.0001.

Although metabolic parameters remained unchanged, the functional efficacy of the training protocol was confirmed by a strength performance assessment. Animals from the ObST and ObSTCr(pic)_3_ groups exhibited significant increases in maximal load capacity in both absolute and relative terms, as well as in Δ strength compared to sedentary Ob and ObCr(pic)_3_ animals (Figure 4A–C). However, Cr(pic)_3_ supplementation did not enhance strength gains beyond those induced by training alone. Thus, while the metabolic phenotype was preserved, ST successfully promoted functional improvement.

Having established systemic and functional adaptations, cardiac structural parameters were evaluated to determine whether the interventions induced morphological remodeling. There were no differences in total heart weight, heart-to-tibia length ratio or resting sarcomere length among the groups, indicating the absence of detectable structural cardiac changes following supplementation and/or training in obese animals (Table 6).

**Figure 5:**
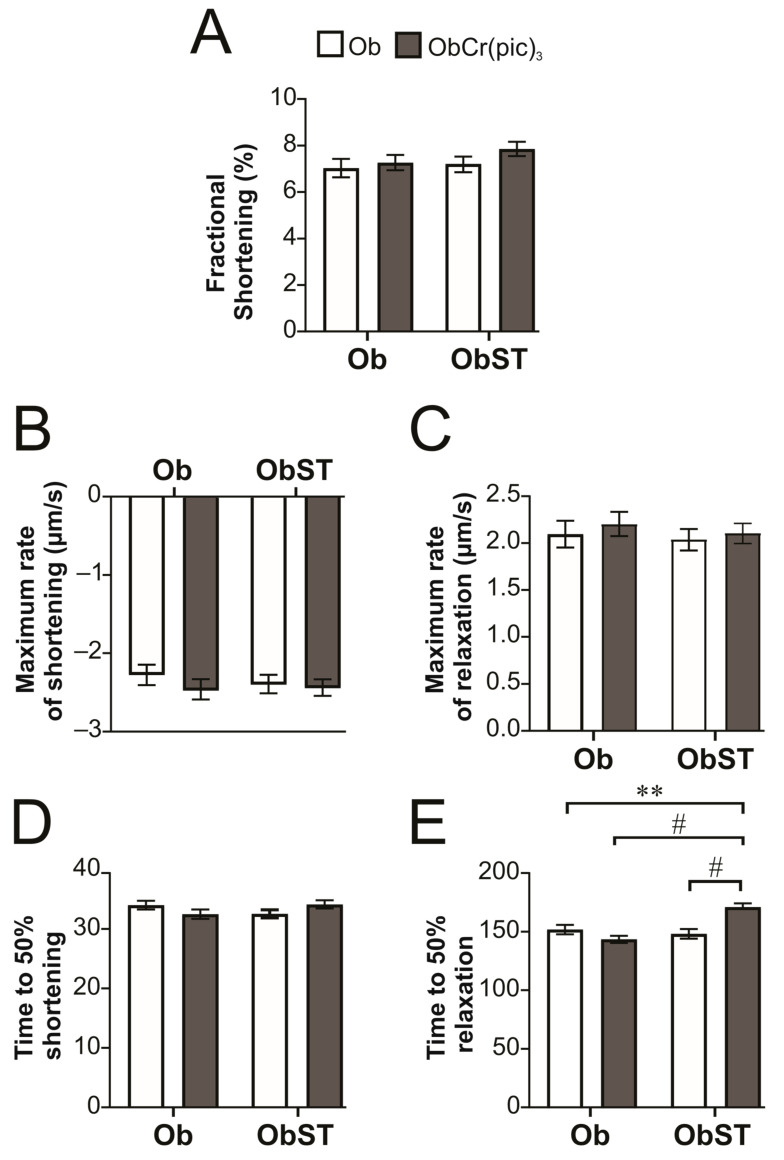
Combination of ST with Cr(pic)_3_ supplementation promotes increased cardiomyocyte relaxation time in obese rats. (**A**) Fractional shortening expressed as % of resting cell length. (**B**) Maximum rate of contraction. (**C**) Maximum rate of relaxation. (**D**) Time to 50% shortening. (**E**) Time to 50% relaxation. Animals per experimental group: Ob: 5 hearts/74 cells; ObST: 6 hearts/101 cells; ObCr(pic)_3_: 4 hearts/69 cells; ObSTCr(pic)_3_: 7 hearts/98 cells. Data are reported as mean ± SEM. Statistical comparisons were performed using two-way ANOVA followed by Bonferroni’s test for multiple comparisons. Data were considered significant when ** *p* = 0.01. # *p* < 0.0001.

Given the preserved cardiac morphology, we examined the contractile properties of cardiomyocytes to assess potential cellular-level alterations. Under electrical stimulation at 1 Hz, fractional shortening, maximal shortening velocity, maximal relaxation velocity and time to 50% shortening were all similar across the groups (Figure 5A–D). Surprisingly, the combination of training with supplementation prolonged relaxation in the ObSTCr(pic)_3_ group compared to their respective controls, Ob, ObCr(pic)_3_ and the ObST groups (Figure 5E). These findings demonstrate that although contractile performance was maintained, the combined intervention unexpectedly delayed cardiomyocyte relaxation kinetics.

## 4. Discussion

This study investigated the combined impact of Cr(pic)_3_ supplementation and ST on metabolic parameters, cardiac morphology and cardiomyocyte contractile function in rats with diet-induced obesity. The main finding was that ST combined with Cr(pic)_3_ supplementation resulted in a prolongation of cardiomyocyte relaxation time without affecting global contractile performance, metabolic profile or cardiac morphology.

An important aspect of diet-induced obesity models is that, unlike in humans, where specific criteria such as body mass index are used, in animal models there is no specific standard or indicator for defining obesity. Thus, the characterization of obesity in rodents is generally based on body mass gain, or an increase in body fat and adiposity index [24,25,26,27,28].

In this context the induction of obesity was effective, as evidenced by the sustained increase in body weight, as well as caloric intake and feed efficiency in the HFD group compared to the SD group. Although the food consumption was lower in this group, the higher energy density of the HFD explains the increased caloric intake and, consequently, the weight gain. Thus, the Ob group showed an increase in visceral, epididymal, and retroperitoneal fat deposits, resulting in a higher adiposity index, which was reflected in the increased body weight of this group when compared to the C group. This finding is consistent with previous studies by our research group, which demonstrated a progressive increase in these parameters in obesity models induced by a high-fat diet compared to a standard diet [23,29,30,31]. Then, these parameters characterized an obesity model.

### Effect of ST and Cr(pic)_3_ Supplementation on Obesity Condition

Despite the absence of significant changes in adiposity, glucose tolerance, insulin resistance, or most lipid parameters, HDL concentrations were modestly higher in the combined intervention group. Neither ST nor Cr(pic)_3_ supplementation alone promoted significant changes in this marker, suggesting that the observed increase may reflect a potential interaction between these interventions rather than an independent effect of either strategy. Changes in HDL concentrations may occur independently of broader metabolic improvements, possibly reflecting selective adaptations in lipid metabolism or lipoprotein transport mechanisms [32,33]. However, the magnitude of this increase was relatively modest and occurred in the absence of consistent metabolic or functional benefits, suggesting that its physiological relevance is unclear.

Although Cr(pic)_3_ is widely publicized as a supplement that can improve glucose metabolism and insulin sensitivity [14,15,34,35], our results did not fully support these claims. No significant improvements in glucose tolerance, insulin resistance or adiposity were observed in the evaluated groups. This suggests that supplementation alone may not be sufficient to promote relevant metabolic adaptations in an obesity model. These findings reinforce the possibility that the metabolic effects of Cr(pic)_3_ depend on the physiological context, the duration of the intervention or its association with other therapeutic strategies.

Chromium (Cr) is an essential mineral that has been used in the form of Cr(pic)_3_, acting on carbohydrate metabolism and improving glucose tolerance [13,14,15]. The estimated and recommended adequate dietary range for Cr in adults is 50–200 μg/day [36]. A range of 200–1000 μg/day has been used in clinical studies [16,37]. Assuming an average body weight of 75 kg, this would refer to an intake between 2.7 and 13.3 μg/kg. The dose of Cr(pic)_3_ used in this study is 80 μg/kg, a dose equal to that previously used by other researchers in studies of Cr(pic)_3_ supplementation in obese rats [18,38]. Thus, the rats in this study received a dose of Cr(pic)_3_ at levels higher than those observed as effective in clinical studies, which can be considered a pharmacological dose. However, caution is needed due to the inconsistency of translational results of Cr(pic)_3_ supplementation. Future studies with longer-duration interventions, varying supplementation doses, and equivalent dosages in humans are necessary to better establish its therapeutic relevance.

As expected, ST significantly increased the absolute and relative load capacity of the trained groups thus confirming the efficacy of the ladder-climbing protocol. These results are consistent with the neural and hypertrophic adaptations that are usually seen in resistance training models [39,40]. Importantly, supplementation with Cr(pic)_3_ did not increase strength gains beyond those achieved through training alone. Therefore, the supplementation of Cr(pic)_3_ did not improve skeletal muscle performance.

The increase in strength alone is already an indicative parameter of health especially in individuals affected by obesity [41]. Studies indicate that high levels of muscle strength are associated with a better cardiometabolic prognosis even in the presence of obesity [42,43]. According to Lima et al. [41] there is a negative correlation between muscle strength and cardiometabolic risk as it is inversely associated with inflammatory markers, insulin resistance and dyslipidemia.

At the cardiac level, no structural remodeling was observed, suggesting that the interventions were not long or intense enough to induce detectable changes in cardiac morphology. This finding is consistent with previous evidence indicating that structural cardiac adaptations necessitate longer or more intense stimuli, particularly in the context of established obesity [30].

The most relevant finding of this study was the alteration in cardiomyocyte relaxation kinetics. Specifically, the combined intervention (ST + Cr(pic)_3_) prolonged the time to 50% relaxation, while other contractile parameters remained unchanged. This suggests a selective impairment in diastolic cellular function rather than a generalized contractile dysfunction. This finding suggests a potential interaction in which supplementation may interfere with exercise-induced cellular adaptations. Importantly, however, fractional shortening, maximum shortening velocity and maximum relaxation velocity remained unchanged. This indicates that the alteration was restricted to cellular relaxation kinetics rather than reflecting a generalized impairment of systolic or diastolic function.

As impaired myocardial relaxation is characteristic of obesity diastolic dysfunction, the prolonged cardiomyocyte relaxation observed in this study could be an early cellular alteration relevant to obesity myocardial relaxation abnormalities [8,44,45]. Myocardial relaxation is primarily regulated by Ca^2+^ reuptake from the sarcoplasmic reticulum, which is mediated by SERCA2a and modulated by phospholamban phosphorylation [46,47]. Alterations in these pathways have been reported in obesity and may also be influenced by exercise or micronutrient supplementation [30,48,49].

Only one study evaluated the effect of Cr(pic)_3_ on the contractile function of cardiomyocytes in the obese condition in which they observed that Cr(pic)_3_ did not alter the contractile function of cardiomyocytes nor the Ca^2+^ transient. A plausible explanation for this discrepancy in our data would be around the treatment time and the dose used in the aforementioned study given that they carried out the treatment for 6 months at a dose of 45 μg/kg/day [16].

The study results have some limitations. First, the molecular mechanisms underlying the observed alterations in cardiomyocyte relaxation were not directly assessed. Future studies investigating calcium-handling proteins and myocardial signaling pathways would help clarify the mechanisms involved. Second, the experimental design focused on isolated cardiomyocyte mechanics, which provides detailed information about cellular function but does not fully capture the complexity of whole-heart physiology.

Despite these limitations, the present study contributes to the understanding of how nutritional supplementation with Cr(pic)_3_ may interact with exercise training to influence cardiomyocyte contractility function in obesity. Further investigations integrating molecular, cellular, and whole-organ approaches will be necessary to determine the physiological significance of these findings and to clarify whether such effects may have implications for cardiovascular health in obesity.

## 5. Conclusions

In conclusion, supplementation with Cr(pic)_3_ or ST alone, or in combination, was not sufficient to induce significant changes in body composition. Although ST improved functional performance, combining it with Cr(pic)_3_ supplementation was associated with prolonged cardiomyocyte relaxation. This suggests that the combination may interfere with cellular mechanisms that regulate diastolic function. These findings emphasize the importance of critically evaluating the use of supplements in conjunction with exercise, particularly in conditions such as obesity that compromise metabolism.

## Figures and Tables

**Table 1:** Resistance training progression protocol.

Week	1st Set	2nd Set	3rd Set	4th Set	5th Set
0	FAMILIARIZATION				
INITIAL MAXIMUM LOAD TEST					
1	50% of ML	75% of ML	90% of ML	100% of ML	100% of ML +30 g
2	50% of ML	75% of ML	90% of ML	100% of ML	100% of ML +30 g
MAXIMUM LOAD TEST					
3	50% of ML	75% of ML	90% of ML	100% of ML	100% of ML +30 g
4	50% of ML	75% of ML	90% of ML	100% of ML	100% of ML +30 g
MAXIMUM LOAD TEST					
5	50% of ML	75% of ML	90% of ML	100% of ML	100% of ML +30 g
6	50% of ML	75% of ML	90% of ML	100% of ML	100% of ML +30 g
MAXIMUM LOAD TEST					
7	50% of ML	75% of ML	90% of ML	100% of ML	100% of ML +30 g
8	50% of ML	75% of ML	90% of ML	100% of ML	100% of ML +30 g
FINAL MAXIMUM LOAD TEST					

Resistance training progression protocol showing the load assigned for each of the five sets per session across the eight weeks of training, with Maximum Load (ML) reassessment performed every two weeks (after Weeks 2, 4, and 6) and at protocol completion (after Week 8). ML: Maximum Load. IMLT: Initial Maximum Load Test. FMLT: Final Maximum Load Test.

**Table 2:** Nutritional characteristics during protocol of obesity induction and maintenance after 14 weeks.

Variables	Groups		
	SD	HFD	*p* Value
Food consumption (g/day)	26.3 ± 0.14	17.7 ± 0.13	<0.0001
Caloric intake (Kcal/day)	76.8 ± 0.4	81.0 ± 0.5	<0.0001
Feed efficiency (%)	2.55 ± 0.04	3.38 ± 0.08	<0.0001
Initial body weight (g)	130 ± 5.4	141 ± 4.4	0.1459
Final body weight (g)	501 ± 13.1	690 ± 30.1	<0.0001
Body weight gains (g)	134 ± 6.4	185 ± 12.6	<0.0014

Nutritional profile of rats in the experimental period: 14 weeks of exposure to obesity. Standard diet (SD, *n* = 59), high-fat diet (HFD, *n* = 59). Data are presented as the mean ± SEM. Statistical comparisons were performed using the two-tailed Student’s *t*-test. Effect of diet (HFD vs SD).

**Table 3:** Nutritional, biochemical, and glycemic profiles.

Variables	Groups	
	C	Ob
IBW (g)	130 ± 18	141 ± 14
FBW (g)	502 ± 44	690 ± 95 *
Body weight gain (g)	371 ± 38	549 ± 87 *
Epididymal (g)	6.44 ± 2.86	13.3 ± 3.9 *
Retroperitoneal (g)	11.9 ± 4.9	34.1 ± 9.7 *
Visceral (g)	7.79 ± 4.10	18.5 ± 5.6 *
Body fat (g)	26.2 ± 11.4	65.9 ± 18.0 *
Adiposity index (%)	5.11 ± 1.87	9.44 ± 1.73 *
Glucose (mg/dL)	87.2 ± 7.94	99.8 ± 9.42 *
Cholesterol (mg/dL)	59.1 ± 10.7	60.8 ± 11.7
LDL (mg/dL)	7.06 ± 1.94	6.00 ± 1.69
HDL (mg/dL)	18.2 ± 4.8	18.8 ± 3.4

Nutritional, biochemical, and glycemic profiles. Animals per experimental group: Control (C. *n* = 11), obese (Ob, *n* = 10). IBW= initial body weight, FBW= final body weight, LDL= low-density lipoprotein, HDL= high-density lipoprotein. Data are presented as the mean ± SEM. Statistical comparisons were performed using a two-tailed Student’s *t*-test. * *p* < 0.05 Ob vs. C.

**Table 4:** Nutritional characteristics and body composition after Cr(pic)_3_ supplementation and ST protocol.

Variables	Groups			
	Ob	ObST	ObCr(pic)_3_	ObSTCr(pic)_3_
Food consumption (g/day)	17.2 ± 0.1	16.9 ± 0.1	17.0 ± 0.1	16.7 ± 0.1
Caloric intake (Kcal/day)	78.7 ± 0.4	77.8 ± 0.9	78.3 ± 0.6	76.8 ± 0.5
Feed efficiency (%)	4.3 ± 0.1	4.3 ± 0.1	4.1 ± 0.1	4.3 ± 0.1
Initial body weight (g)	620.9 ± 24.8	612 ± 21.6	599 ± 13	617.1 ± 17.8
Final body weight (g)	690.4 ± 30.1	688.2 ± 21.2	661.5 ± 14.9	666.2 ± 20.1
Epididymal fat (g)	13.3 ± 1.2	12.6 ± 1.07	12.3 ± 1.2	12.2 ± 0.7
Retroperitoneal fat (g)	34.1 ± 3.05	37.8 ± 2.6	33.9 ± 3.6	30.9 ± 1.5
Visceral fat (g)	18.4 ± 1.7	18.8 ± 1.2	17.05 ± 1.7	16.6 ± 1.7
Body fat (g)	65.9 ± 5.6	69.3 ± 4.4	63.2 ± 6.2	59.8 ± 3.5
Adiposity index (%)	9.4 ± 0.5	10.01 ± 0.4	9.4 ± 0.8	8.9 ± 0.3

Nutritional characteristics and body composition of rats. Animals per experimental group: obese (Ob, *n* = 10), obese strength training (ObST, *n* = 11), obese supplementation with chromium picolinate (ObCr(pic)_3_, *n* = 11), and obese combination of both (ObSTCr(pic)_3_, *n* = 11). Data are reported as mean ± SEM. Statistical comparisons were performed using two-way ANOVA followed by Bonferroni’s test for multiple comparisons.

**Table 5:** Profile of comorbidities associated with obesity after Cr(pic)_3_ supplementation and ST protocol.

Variables	Groups			
	Ob	ObST	ObCr(pic)_3_	ObSTCr(pic)_3_
Glucose (mg/dL)	99.7 ± 3.1	97 ± 2.8	96.8 ± 3.1	95.3 ± 2.3
Insulin (ng/mL)	1.6 ± 0.4	2.4 ± 0.09	2.5 ± 0.1	2.6 ± 0.02
Homa-IR	36.2 ± 1	30.3 ± 6.5	42 ± 5.8	40.3 ± 3.1
Cholesterol (mg/dL)	60.8 ± 4.1	65.7 ± 3.5	66.5 ± 4.01	67.3 ± 1.3
LDL (mg/dL)	6.0 ± 0.5	6.8 ± 0.5	6.9 ± 1	6.2 ± 0.2
HDL (mg/dL)	18.7 ± 1.2	22.0 ± 0.2	19.8 ± 1.1	22.9 ± 0.5 **
Triglycerides (mg/dL)	16.6 ± 1	21.5 ± 3.6	24.0 ± 1.7	16.8 ± 1

Profile of comorbidities after supplementation and strength training protocol. Animals per experimental group: obese (Ob, *n* = 10), obese strength training (ObST, *n* = 11), obese supplementation with chromium picolinate (ObCr(pic)_3_, *n* = 11), and obese combination of both (ObSTCr(pic)_3_, *n* = 11). Data are reported as mean ± SEM. Statistical comparisons were performed using two-way ANOVA followed by Bonferroni’s test for multiple comparisons. ** *p* < 0.01 Ob vs. ObSTCr(pic)_3_.

**Table 6:** Cardiac hypertrophy index after Cr(pic)_3_ supplementation and ST protocol.

Variables	Groups			
	Ob	ObST	ObCr(pic)_3_	ObSTCr(pic)_3_
Heart (g)	2.6 ± 0.1	2.5 ± 0.06	2.6 ± 0.2	2.3 ± 0.06
Heart/Tibia (g/cm)	0.5 ± 0.02	0.5 ± 0.01	0.5 ± 0.04	0.5 ± 0.009
Sarcomere length (µm)	1.7 ± 0.007	1.7 ± 0.008	1.7 ± 0.009	1.7 ± 0.008

Cardiac hypertrophy index after supplementation and strength training protocol. Animals per experimental group: obese (Ob, *n* = 5), obese strength training (ObST, *n* = 6), obese supplementation with chromium picolinate (ObCr(pic)_3_, *n* = 4), and obese combination of both (ObSTCr(pic)_3_, *n* = 7). Data are reported as mean ± SEM. Statistical comparisons were performed using two-way ANOVA followed by Bonferroni’s test for multiple comparisons.

## Data Availability

The datasets used and/or analyzed during the current study are available from the corresponding author upon request.

## Abbreviations

The following abbreviations are used in this manuscript:

SD	Standard diet
HFD	High-fat diet
ST	Strength training
Cr(pic)_3_	Chromium Picolinate

## References

[1] Eckel R.H., Barouch W.W., Ershow A.G. (2002). Report of the National Heart, Lung, and Blood Institute-National Institute of Diabetes and Digestive and Kidney Diseases Working Group on the Pathophysiology of Obesity-Associated Cardiovascular Disease. Circulation.

[2] Carvajal K., Balderas-Villalobos J., Bello-Sanchez M.D., Phillips-Farfán B., Molina-Muñoz T., Aldana-Quintero H., Gómez-Viquez N.L. (2014). Ca^2+^ Mishandling and Cardiac Dysfunction in Obesity and Insulin Resistance: Role of Oxidative Stress. Cell Calcium.

[3] Carroll J.F., Zenebe W.J., Strange T.B. (2006). Cardiovascular Function in a Rat Model of Diet-Induced Obesity. Hypertension.

[4] Lima-Leopoldo A.P., Leopoldo A.S., Sugizaki M.M., Bruno A., Nascimento A.F., Luvizotto R.A.M., Oliveira Júnior S.A.d., Castardeli E., Padovani C.R., Cicogna A.C. (2011). Myocardial Dysfunction and Abnormalities in Intracellular Calcium Handling in Obese Rats. Arq. Bras. Cardiol..

[5] Coelho P.M., Simmer L.M., da Silva D.S., dos Santos M.C., Kitagawa R.R., Pezzin M.F., Correa C.R., Leite J.G., Leopoldo A.S., Lima-Leopoldo A.P. (2023). Type 2 Diabetes Mellitus in Obesity Promotes Prolongation of Cardiomyocyte Contractile Function, Impaired Ca^2+^ Handling and Protein Carbonylation Damage. J. Diabetes Its Complicat..

[6] Sequeira V., Theisen J., Ermer K.J., Oertel M., Xu A., Weissman D., Ecker K., Dudek J., Fassnacht M., Nickel A. (2025). Semaglutide Normalizes Increased Cardiomyocyte Calcium Transients in a Rat Model of High Fat Diet-Induced Obesity. ESC. Heart Fail..

[7] Wen X., Zhang B., Wu B., Xiao H., Li Z., Li R., Xu X., Li T. (2022). Signaling Pathways in Obesity: Mechanisms and Therapeutic Interventions. Signal Transduct. Target. Ther..

[8] Liu J., Li J., Xia C., He W., Li X., Wang Y., Shen S., Tong N., Peng L. (2024). Diastolic Dysfunction in Adults with Uncomplicated Obesity Evaluated with Left Atrial and Left Ventricular Tissue Tracking and Ventricular Volume-Time Curve: A Prospective Cardiac Magnetic Resonance Study. Quant. Imaging Med. Surg..

[9] Fu Z., Wang Y., Wang Y., Shi S., Li Y., Zhang B., Wu H., Song Q. (2024). Linking Abnormal Fat Distribution with HFpEF and Diastolic Dysfunction: A Systematic Review, Meta-Analysis, and Meta-Regression of Observational Studies. Lipids Health Dis..

[10] Pedersen B.K. (2006). The Anti-Inflammatory Effect of Exercise: Its Role in Diabetes and Cardiovascular Disease Control. Essays Biochem..

[11] Nazir A., Heryaman H., Juli C., Ugusman A., Martha J.W., Moeliono M.A., Atik N. (2024). Resistance Training in Cardiovascular Diseases: A Review on Its Effectiveness in Controlling Risk Factors. Integr. Blood Press. Control.

[12] Ahmadiasl N., Najafipour H., Soufi F.G., Jafari A. (2012). Effect of short- and long-term strength exercise on cardiac oxidative stress and performance in rat. J. Physiol. Biochem..

[13] Anton S.D., Morrison C.D., Cefalu W.T., Martin C.K., Coulon S., Geiselman P., Han H., White C.L., Williamson D.A. (2008). Effects of Chromium Picolinate on Food Intake and Satiety. Diabetes Technol. Ther..

[14] Tian H., Guo X., Wang X., He Z., Sun R., Ge S., Zhang Z. (2013). Chromium Picolinate Supplementation for Overweight or Obese Adults. Cochrane Database Syst. Rev..

[15] Peng M., Yang X. (2015). Controlling Diabetes by Chromium Complexes: The Role of the Ligands. J. Inorg. Biochem..

[16] Dong F., Yang X., Sreejayan N., Ren J. (2007). Chromium (d-Phenylalanine)3 Improves Obesity-Induced Cardiac Contractile Defect in Ob/Ob Mice. Obesity.

[17] AL-Rasheed N.M., Attia H.A., Mohamed R.A., Al-Rasheed N.M., Al-Amin M.A. (2014). Preventive Effects of Selenium Yeast, Chromium Picolinate, Zinc Sulfate and Their Combination on Oxidative Stress, Inflammation, Impaired Angiogenesis and Atherogenesis in Myocardial Infarction in Rats. J. Pharm. Pharm. Sci..

[18] Sahin K., Tuzcu M., Orhan C., Gencoglu H., Ulas M., Atalay M., Sahin N., Hayirli A., Komorowski J.R. (2012). The Effects of Chromium Picolinate and Chromium Histidinate Administration on NF-κB and Nrf2/HO-1 Pathway in the Brain of Diabetic Rats. Biol. Trace Elem. Res..

[19] Speretta G.F.F., Rosante M.C., Duarte F.O., Leite R.D., Lino A.D.S., Andre R.A., Silvestre J.G.O., de Araujo H.S.S., Duarte A.C.G.O. (2012). The Effects of Exercise Modalities on Adiposity in Obese Rats. Clinics.

[20] Leite R.D., Durigan R.d.C.M., Lino A.D.d.S., Campos M.V.d.S., Souza M.d.G., Selistre-de-Araújo H.S., Bouskela E., Kraemer-Aguiar L.G. (2013). Resistance Training May Concomitantly Benefit Body Composition, Blood Pressure and Muscle MMP-2 Activity on the Left Ventricle of High-Fat Fed Diet Rats. Metab.-Clin. Exp..

[21] Cordeiro J.P., Silva D.S.d., Torezani-Sales S., Madureira A.R., Claudio E.R.G., Bocalini D.S., Lima-Leopoldo A.P., Leopoldo A.S. (2022). Resistance to Obesity Prevents Obesity Development without Increasing Spontaneous Physical Activity and Not Directly Related to Greater Metabolic and Oxidative Capacity. PLoS ONE.

[22] Mendes B.F., Costa-Pereira L.V., de Andrade J.A., Magalhães C.O.D., de Pereira R.R.S., Esteves E.A., Cassilhas R.C., Andrade E.F., Gripp F., de Magalhães F.C. (2022). Superior Cardiometabolic and Cellular Adaptive Responses to Multiple versus Single Daily Sessions of High-Intensity Interval Training in Wistar Rats. Sci. Rep..

[23] dos Santos M.C., da Silva D.S., Cordeiro J.P., Domingos L.F., Gomes E.H.S., Nogueira B.V., Bocalini D.S., Lima-Leopoldo A.P., Leopoldo A.S. (2024). High-intensity Interval Training Improves Cardiomyocyte Contractile Function and Myofilament Sensitivity to Intracellular Ca^2+^ in Obese Rats. Exp. Physiol..

[24] Hariri N., Thibault L. (2010). High-fat diet-induced obesity in animal models. Nutr. Res. Rev..

[25] Gasparini P.V.F., Matias A.M., Torezani-Sales S., Kobi J.B.B.S., Siqueira J.S., Corrêa C.R., Lima-Leopoldo A.P., Leopoldo A.S. (2021). High-Fat and Combined High-Fat and Sucrose Diets Promote Cardiac Oxidative Stress Independent of Nox2 Redox Regulation and Obesity in Rats. Cell. Physiol. Biochem..

[26] Jin D., Xu Y., Mei X., Meng Q., Gao Y., Li B., Tu Y. (2013). Antiobesity and lipid lowering effects of theaflavins on high-fat diet induced obese rats. J. Funct. Foods.

[27] Kobi J.B.B.S., Matias A.M., Gasparini P.V.F., Torezani-Sales S., Madureira A.R., da Silva D.S., Correa C.R., Garcia J.L., Haese D., Nogueira B.V. (2023). High-fat, high-sucrose, and combined high-fat/high-sucrose diets effects in oxidative stress and inflammation in male rats under presence or absence of obesity. Physiol. Rep..

[28] Matias A.M., Coelho P.M., Marques V.B., dos Santos L., de Assis A.L.E.M., Nogueira B.V., Lima-Leopoldo A.P., Leopoldo A.S. (2020). Hypercaloric diet models do not develop heart failure, but the excess sucrose promotes contractility dysfunction. PLoS ONE.

[29] Cordeiro J.P., Silva V.L.d., Campos D.H., Cicogna A.C., Leopoldo A.S., Lima-Leopoldo A.P. (2021). Isolated Obesity Resistance Condition or Associated with Aerobic Exercise Training Does Not Promote Cardiac Impairment. Braz. J. Med. Biol. Res..

[30] Melo A.B., Damiani A.P.L., Coelho P.M., de Assis A.L.E.M., Nogueira B.V., Ferreira L.G., Leite R.D., Ribeiro Júnior R.F., Lima-Leopoldo A.P., Leopoldo A.S. (2020). Resistance Training Promotes Reduction in Visceral Adiposity without Improvements in Cardiomyocyte Contractility and Calcium Handling in Obese Rats. Int. J. Med. Sci..

[31] Santos K.C.C., Domingos L.F., Nunes F.M., Simmer L.M., Cordeiro E.R., Filetti F.M., Bocalini D.S., Corrêa C.R., Lima-Leopoldo A.P., Leopoldo A.S. (2024). Capsinoids Increase Antioxidative Enzyme Activity and Prevent Obesity-Induced Cardiac Injury without Positively Modulating Body Fat Accumulation and Cardiac Oxidative Biomarkers. Nutrients.

[32] Trajkovska K.T., Topuzovska S. (2017). High-Density Lipoprotein Metabolism and Reverse Cholesterol Transport: Strategies for Raising HDL Cholesterol. Anatol. J. Cardiol..

[33] Pownall H.J., Rosales C., Gillard B.K., Gotto A.M. (2021). High-density lipoproteins, reverse cholesterol transport and atherogenesis. Nat. Rev. Cardiol..

[34] Marmett B., Nunes R.B. (2016). Effects of Chromium Picolinate Supplementation on Control of Metabolic Variables: A Systematic Review. J. Food Nutr. Res..

[35] Costello R.B., Dwyer J.T., Merkel J.M. (2019). Chromium Supplements in Health and Disease. The Nutritional Biochemistry of Chromium (III).

[36] Jain S.K., Rains J.L., Croad J.L. (2007). Effect of chromium niacinate and chromium picolinate supplementation on lipid peroxidation, TNF-α, IL-6, CRP, glycated hemoglobin, triglycerides, and cholesterol levels in blood of streptozotocin-treated diabetic rats. Free. Radic. Biol. Med..

[37] Staniek H., Rhodes N.R., Di Bona K.R., Deng G., Love S.T., Pledger L.A., Blount J., Gomberg E., Grappe F., Cernosek C. (2013). Comparison of Tissue Metal Concentrations in Zucker Lean, Zucker Obese, and Zucker Diabetic Fatty Rats and the Effects of Chromium Supplementation on Tissue Metal Concentrations. Biol. Trace Elem. Res..

[38] Komorowski J.R., Tuzcu M., Sahin N., Juturu V., Orhan C., Ulas M., Sahin K. (2012). Chromium Picolinate Modulates Serotonergic Properties and Carbohydrate Metabolism in a Rat Model of Diabetes. Biol. Trace. Elem. Res..

[39] Padilha C.S., Cella P.S., Ribeiro A.S., Voltarelli F.A., Testa M.T.J., Marinello P.C., Iarosz K.C., Guirro P.B., Deminice R. (2019). Moderate vs High-Load Resistance Training on Muscular Adaptations in Rats. Life Sci..

[40] Krause Neto W., Silva W., Oliveira T., Vilas Boas A., Ciena A., Caperuto É.C., Gama E.F. (2024). Ladder-Based Resistance Training with the Progression of Training Load Altered the Tibial Nerve Ultrastructure and Muscle Fiber Area without Altering the Morphology of the Postsynaptic Compartment. Front. Physiol..

[41] De Lima T.R., González-Chica D.A., Sui X., Silva D.A.S. (2022). The Independent and Joint Associations among Muscle Strength, Abdominal Obesity and Cardiometabolic Variables among Adults. Eur. J. Sport Sci..

[42] Carbone S., Kirkman D.L., Garten R.S., Rodriguez-Miguelez P., Artero E.G., Lee D., Lavie C.J. (2020). Muscular Strength and Cardiovascular Disease: An Updated State-Of-The-Art Narrative Review. J. Cardiopulm. Rehabil. Prev..

[43] De Lima T.R., Martins P.C., Guerra P.H., Silva D.A.S. (2021). Muscular Strength and Cardiovascular Risk Factors in Adults: A Systematic Review. Phys. Sportsmed..

[44] Rayner J.J., Banerjee R., Holloway C.J., Lewis A.J.M., Peterzan M.A., Francis J.M., Neubauer S., Rider O.J. (2018). The Relative Contribution of Metabolic and Structural Abnormalities to Diastolic Dysfunction in Obesity. Int. J. Obes..

[45] Russo C., Jin Z., Homma S., Rundek T., Elkind M.S.V., Sacco R.L., Di Tullio M.R. (2011). Effect of Obesity and Overweight on Left Ventricular Diastolic Function. JACC.

[46] Vittone L., Mundina-Weilenmann C., Mattiazzi A. (2008). Phospholamban Phosphorylation by CaMKII under Pathophysiological Conditions. Front. Biosci..

[47] Kranias E.G., Hajjar R.J. (2012). Modulation of Cardiac Contractility by the Phopholamban/SERCA2a Regulatome. Circ. Res..

[48] Leopoldo A.S., Lima-Leopoldo A.P., Sugizaki M.M., Nascimento A.F., de Campos D.H.S., Luvizotto R.A.M., Castardeli E., Alves C.A.B., Brum P.C., Cicogna A.C. (2011). Involvement of L-Type Calcium Channel and Serca2a in Myocardial Dysfunction Induced by Obesity. J. Cell. Physiol..

[49] Abdel-Hady E.A. (2024). Chromium Picolinate Supplementation Improves Cardiac Performance in Hypoxic Rats. Acta Cardiol..

